# The Process Analysis Method of SAR Target Recognition in Pre-Trained CNN Models

**DOI:** 10.3390/s23146461

**Published:** 2023-07-17

**Authors:** Tong Zheng, Jin Li, Hao Tian, Qing Wu

**Affiliations:** 1 School of Artificial Intelligence, Beijing Technology and Business University, Beijing 100048, China; 20211206@btbu.edu.cn (T.Z.); 20201005@btbu.edu.cn (H.T.); 2 Heilongjiang Province Key Laboratory of Laser Spectroscopy Technology and Application, Harbin University of Science and Technology, Harbin 150006, China; wuqing@buaa.edu.cn

**Keywords:** synthetic aperture radar (SAR), convolutional neural network (CNN), process analysis, image filtering, MSTAR

## Abstract

Recently, attention has been paid to the convolutional neural network (CNN) based synthetic aperture radar (SAR) target recognition method. Because of its advantages of automatic feature extraction and the preservation of translation invariance, the recognition accuracies are stronger than traditional methods. However, similar to other deep learning models, CNN is a “black-box” model, whose working process is vague. It is difficult to locate the decision reasons. Because of this, we focus on the process analysis of a pre-trained CNN model. The role of the processing to feature extraction and final recognition decision is discussed. The discussed components of CNN models are convolution, activation function, and full connection. Here, the convolution processing can be deemed as image filtering. The activation function provides a nonlinear element of processing. Moreover, the fully connected layers can also further extract features. In the experiment, four classical CNN models, i.e., AlexNet, VGG16, GoogLeNet, and ResNet-50, are trained by public MSTAR data, which can realize ten-category SAR target recognition. These pre-trained CNN models are processing objects of the proposed process analysis method. After the analysis, the content of the SAR image target features concerned by these pre-trained CNN models is further clarified. In summary, we provide a paradigm to process the analysis of pre-trained CNN models used for SAR target recognition in this paper. To some degree, the adaptability of these models to SAR images is verified.

## 1. Introduction

The synthetic aperture radar (SAR) is an active sensor, which can obtain observation information by transmitting electromagnetic signals and receiving echo signals. Compared with other sensors, the SAR has the advantage of all-day and all-weather observations. In addition, the coherent imaging mechanism and multiple polarization modes of the SAR can increase the observation information. In reality, applications of the SAR are based on imaging results. We can extract the regions of interest in SAR images to achieve the correct description and expression of the target [1]. From then on, several related studies are developed, including Automatic Target Recognition (ATR). The application of SAR images in the field of marine monitoring, geological exploration, etc. has been promoted [2,3,4]. In contrast to traditional SAR image interpretability methods, deep learning can extract inherent patterns from the large amounts of data. With the advantages of hierarchical extraction, layer-by-layer extraction, and end-to-end spontaneous learning, the deep learning method has become a hot topic in many fields. For example, deep neural network regression for automated retinal layer segmentation in optical coherence tomography images [5], immune extremum region for floating pollutant image target extraction algorithm [6], immune neural network for a fault detection algorithm for pipeline insulation layer [7], etc. In deep learning methods, the convolutional neural network (CNN) is first used for handwritten character image recognition. Then, it is extended to solve the problem of target detection, face detection, target tracking, face recognition, video classification, edge detection, image segmentation, and so on [8,9]. Moreover, it combines the ideas of the local field of perception, pooling, and weights sharing. It can dig local relevant features and maintain translation invariance in images. Hence, CNN has become an important branch of research in SAR target recognition methods [10,11].

However, the imaging mechanisms of SAR and optical images are different. There are some specific problems in CNN-based SAR target recognition, i.e., improvements in accuracy [12,13,14], small sample quantity [15,16,17,18], strong speckle noise [19,20,21,22], etc. For example, to improve the accuracy and highlight important features of SAR targets, ref. [12] placed a full convolutional module, which includes channel attention and space attention, into classical CNN models. Moreover, a small SAR sample quantity may cause an overfitting problem for complex models. Therefore, [18] provides a method based on contrast learning and false labeling, which can reduce the need for large samples in model training to a large extent. According to the speckle noise problem, aiming to improve the robustness of the model, there are some studies based on the joint connection of despeckling and recognition CNN models [15], which is similar to our team.

Because of the “black-box” characteristic of the CNN, its work process is vague. Moreover, it is difficult to locate the key reason of decision. This is the main reason damaging the practical application of the CNN model. According to this problem, the interpretability of the CNN has received widespread attention at home and abroad. In recent years, there have been some research achievements. For example, ref. [23] proposes a CNN interpretability analysis method for text classification, which focuses on the discriminant results of multi-classification text and multi-label classification tasks through backtracking analysis of the model prediction results. Moreover, to realize image denoising, ref. [24] use discrete cosine transform (DCT) to replace the shallow layer processing of the CNN. It improves the reasonability of the CNN processing. According to the method of [25], in the learning process, each explainable filter in the deep layer is automatically assigned a specific category of targets, which realizes a better understanding of the CNN coding logic by explicit knowledge representation. These studies serve as the foundation for the interpretability research on the CNN.

Regarding the SAR target recognition task, the CNN interpretability study is at a preliminary stage. The related studies are direct applications of classical methods, but do not pay attention to SAR image characteristics. For example, layer-wise relevance propagation (LRP) [26] is used to show the relevance score of the SAR images and output labels [27]. In addition, gradient-weighted class activation mapping (Grad-CAM) [28], local interpretable model-agnostic explanations (LIME) [29], and shapley additive explanations (SHAP) [30] are applied to evaluate feature correlations for a seven-layer pre-trained CNN model in the SAR target recognition task [31]. The experiments show that the target recognition decision of the CNN model is guided by the target region in the SAR image rather than the background clutter region. It is obvious that the classical interpretability analysis method is applied into the SAR image target recognition task, while ignoring the influences of the SAR image characteristics in these studies. As a result, the adaptability of the pre-trained CNN model is difficult to guarantee. According to this problem, we pay attention to the process analysis of pre-trained CNN models used for SAR target recognition. Here, the key processing links of the CNN, i.e., convolution, activation, and full connection, are of concern in this paper. More importantly, we provide a paradigm for the process analysis of pre-trained CNNs used for SAR target recognition.

The remainder of our paper is structured as follows. Section 2 describes the theoretical foundation of SAR images, including speckle noise and multi-view characteristics. Then, Section 3 outlines the characteristics of classical CNN models and the effect of speckle noise on SAR target recognition. In the Section 4, we provide the process analysis method of the pre-trained CNN for SAR target recognition. The experiments and discussions are carried out in Section 5. Finally, the research content of this paper is summarized in the Section 6.

## 2. Related Works

SAR imaging reflects the intensity spatial distribution of complex scattering echoes of targets and background in the irradiated area. Moreover, the imaging results are closely related to the radar operating state, radar operating wavelength, incidence angle, polarization mode, target structure, target attitude, target environment, etc. Hence, if imaging conditions are different for one target, the corresponding SAR images will vary. Here, due to the SAR imaging mechanism, the inherent noise, namely, speckle noise, will blur the edge of SAR images. It further improves the difficulty of SAR image interpretation.

### 2.1. Speckle Noise Characteristic of SAR Images

In the process of observation, one resolution unit includes the information of a surface target with a certain area in the observation scene, which is obtained by several scatter echoes. The total echo is generated by the incoherent superposition of these scatter echoes. Under the fully developed speckle hypothesis, based on the central limit theorem [32], the synthetic radar echo can be represented as a complex signal:

(1) $$Ae^{j\phi }=z_{1}+jz_{2},$$

where A and ϕ are the amplitude and phase of the signal, respectively. Moreover, the complex signal can be represented by real ($z_{1}$) and imaginary ($z_{2}$) parts, as in Equation (1). Here, $z_{1}$ and $z_{2}$ both obey Gaussian distributions with means of 0 and variances of σ/2. They are independent and identically distributed. The joint probability density function (PDF) can be shown as:

(2) $$p_{z_{1},z_{2}}\left(z_{1},z_{2}\right)=\frac{1}{\pi \sigma }e^{-\frac{z_{1}^{2}+z_{2}^{2}}{\sigma }},$$

Moreover, the signal intensity obeys an exponential distribution, whose PDF is:

(3) $$p_{I}\left(I\right)=\frac{I}{\sigma }e^{-\frac{I}{\sigma }},$$

where *I* is the intensity. In the SAR system, *L* independent measurements are usually obtained for one observation sample, and then incoherent average processing is carried out to obtain the final measurement result. The PDF of the final measurement result can be acquired by *L* convolutions of Equation (3) as:

(4) $$p_{\left.I_{L}\right|\sigma }\left(\left.I_{L}\right|\sigma \right)=\frac{1}{\Gamma \left(L\right)}{\left(\frac{L}{\sigma }\right)}^{L}I_{L}^{L-1}e^{-\frac{LI_{L}}{\sigma }},$$

where $\Gamma \left(\cdot \right)$ is the gamma function. It is obvious that the final measurement results obey the gamma distribution whose shape parameter and scale parameter are *L* and σ/L, respectively. When the pixel spacing is close to the radiation resolution, the SAR intensity data can be modeled in the multiplicative form [32,33], which can be shown as:

(5) $$I_{unit}=R_{t}\cdot u,$$

where $R_{t}$ is backscattering and *u* is multiplicative speckle noise not related with $R_{t}$. Moreover, the speckle noise usually obeys the gamma distribution with α=β=L [33]. Hence, we substitute σ=1 into Equation (4) and get the result:

(6) $$p\left(u\right)=\frac{L^{L}\cdot u^{L-1}}{\Gamma \left(L\right)}e^{-Lu},$$

**Figure 1:**
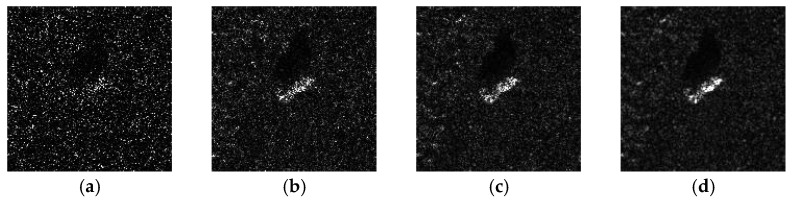
Comparison of the SAR images of the same BMP2 target with different levels of speckled noise: (**a**) *L* = 0.2; (**b**) *L* = 1; (**c**) *L* = 5; (**d**) original SAR image.

According to Equation (6), we can find that the faster the gray change of the SAR image, the faster the speckle noise various. Taking one BMP2 target SAR image in the public MSTAR dataset as an example, speckle noises with different *L* are infused, respectively. The imaging comparison results are shown in Figure 1. It is obvious that the smaller the *L*, the worse the image quality.

### 2.2. Multi-View Characteristics of SAR Images

**Figure 2:**
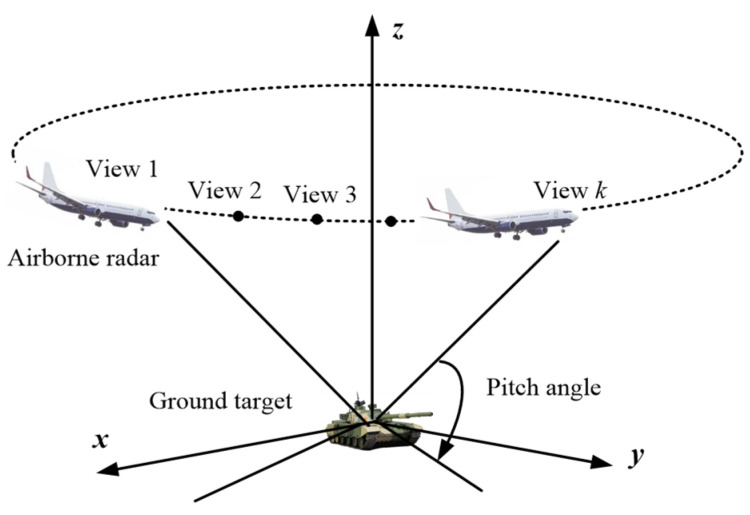
SAR multi-view information acquisition diagram.

The performance of the SAR ATR in practical applications is affected by many factors, such as the battlefield environment, target characteristics, imaging parameters, etc. Here, the target characteristics of single-view SAR will fluctuate with the change of view. In addition, in SAR image processing, it is assumed that the target scattering characteristics remain unchanged during azimuth coherent accumulation, and that the scattering information related to the target viewing angle is lost in single-view SAR images. On the contrary, the multi-view SAR images of the same target contain more abundant classification and recognition information [34]. As a new way of information detection and perception, multi-view SAR target recognition technology obtains multiple images of targets from different perspectives based on the SAR platform. It uses the scattering characteristics of different perspectives to discriminate the category of targets, which has its unique advantages compared with single-view SAR target recognition [35,36]. The multi-view information collection of the same target is shown in Figure 2. Hence, in practice, it is a more reliable method to observe the same target from multiple views and obtain useful information.

It is obvious that although we want to monitor one target, the SAR imaging results vary due to the different views. This is also a big difficulty in SAR image target recognition.

## 3. The Characteristics of the Classical CNN Models and the Effect of Speckle Noise on the Accuracy

The classical CNN models include AlexNet [37], GoogLeNet, Visual Geometry Group (VGG) [38], Residual Network (ResNet) [39], etc. There are some characteristics of these models.

Firstly, AlexNet is composed of five convolutional layers, three pooling layers, and three fully connected layers. Compared with the former CNN models, AlexNet has deep convolutional layers and a bigger receptive field to fit large datasets. It is the bridge between shallow and deep neural networks. Secondly, compared with AlexNet, the convolution kernels of the VGG are small, which can effectively reduce the computational cost. In the meantime, to ensure that the receptive field is big enough, the network is deepened. The VGG11, VGG13, VGG16, and VGG19 are introduced in [38]. In this paper, we pay attention to VGG16. Moreover, the main characteristic of GoogLeNet is the “Inception” module. Namely, the multi-level features are concatenated and input into the next layer. The multi-scale features are extracted by the “Inception” module. In addition, the 1 × 1 convolution kernels are introduced, which are used for dimension reduction. Finally, to avoid the problem of gradient dispersion or gradient explosion caused by layer deepening, the batch normalization (BN) layer is introduced in ResNet. It can weaken the strong connection between each layer through the interlayer connection of feature maps. Then, it is realized that it mitigates network degradation. ResNet with 18, 34, 50, 101, and 152 layers has been introduced. In this paper, we just analyze ResNet-50.

In order to analyze the effect of speckle noise on recognition accuracy, we pre-trained the aforementioned models with SAR images in the public MSTAR dataset. There are 10 types of targets in this dataset. Here, the testing SAR images are synthetically generated from raw SAR images and simulated speckle noise. According to Section 2.1, we can adjust the SAR image quality by changing the value of *L*. Here, L→∞ represents the test images, which are raw SAR images. The test results of the four classical models are shown in Table 1.

According to the last row of Table 1, we can find that the accuracies of these models are up to 0.97 for raw SAR images, representing the strong recognition ability of classical models. However, it is obvious that no matter which model is used, the accuracy goes down following the decrease in *L*. From the analysis of Section 2.1, SAR image quality deteriorative when *L* is small. The recognition difficulty is enhanced in this case. Accordingly, the recognition results deteriorate. This is the effect of speckle noise on SAR target recognition.

## 4. Process Analysis Method of Pre-Trained CNN Models

**Figure 3:**
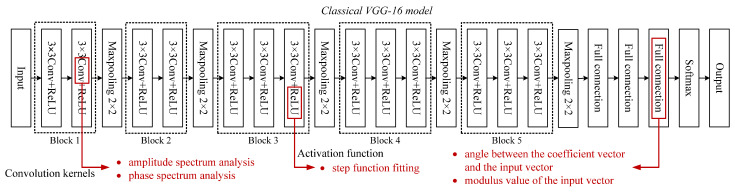
The concrete study content of this paper.

In this section, we will show the process analysis method of the CNN model. It is displayed based on the composition of the CNN model. Hence, we provide analysis methods in convolution, activation, and full connection, respectively. The concrete study content is shown in Figure 3 (red words).

### 4.1. Convolution Processing

Because the convolution processing is equivalent to a zero phase shift filter, we pay attention to the convolution kernels in the frequency domain. Therefore, from the perspective of the frequency domain, the effect of feature extraction on SAR images by convolution processing in the CNN is analyzed. Here, SAR images are presented as discrete images and their spectrum can be obtained by two-dimensional discrete Fourier transform (2D-DFT) [40]. Specifically, assuming the size of the spatial SAR image *f*(*x*,*y*) is *A* × *B*, the 2D-DFT processing result of *f*(*x*,*y*) can be expressed as:

(7) $$F\left(u,v\right)=F\left[f\left(x,y\right)\right]=\frac{1}{N^{2}}\sum_{x=0}^{P-1}\sum_{y=0}^{Q-1}f\left(x,y\right)\cdot e^{-j2\pi \left(\frac{ux}{N}+\frac{vy}{N}\right)}=\left|F\left(u,v\right)\right|\cdot e^{j\varphi \left(u,v\right)},$$

where F is the symbolic representation of 2D-DFT. $\left|F\left(u,v\right)\right|$ and $\varphi \left(u,v\right)$ are amplitude and phase spectra, respectively. They can be shown as:

(8) $$\left|F\left(u,v\right)\right|=\sqrt{R^{2}\left(u,v\right)+I^{2}\left(u,v\right)},$$

(9) $$\varphi \left(u,v\right)=\arctan\left[\frac{I\left(u,v\right)}{R\left(u,v\right)}\right],$$

where $R\left(u,v\right)$ and $I\left(u,v\right)$ are real and imaginary parts of $F\left(u,v\right)$, respectively. In general, centralized processing is necessary to the spectrum obtained. Therefore, the center point of the spectrum corresponds to the zero frequency point. Moreover, the corresponding frequency gradually increases with the increase in the distance from the zero frequency point.

The feature extraction process of convolution processing in CNN models can be analyzed based on the convolution theorem. A discrete convolution calculation is carried out between the raw SAR image *f*(*x*,*y*) and convolution kernel *k*(*x*,*y*) in the CNN. After conversion of the frequency domain, the result can be expressed as:

(10) $$f\left(x,y\right)⊗k\left(x,y\right)\Leftrightarrow F\left(u,v\right)K\left(u,v\right),$$

According to the convolution theorem, convolution processing in the space domain can be equivalent to dot product processing in the frequency domain. Therefore, the convolution processing in CNN can be deemed as the dot product processing of the input SAR image spectrum and the convolution kernel spectrum in the frequency domain. This is the fundamental to frequency domain analysis of convolution processing. Here, the amplitude spectrum of the result after convolution processing can be shown as:

(11) $$\left|F\left(u,v\right)\cdot K\left(u,v\right)\right|=\left|\left|F\left(u,v\right)\right|e^{j\varphi \left(u,v\right)}\cdot \left|K\left(u,v\right)\right|e^{j\varphi_{k}\left(u,v\right)}\right|=\left|F\left(u,v\right)\right|\cdot \left|K\left(u,v\right)\right|,$$

where $\left|F\left(u,v\right)\right|$ and $\left|K\left(u,v\right)\right|$ are amplitude spectrums of *f*(*x*,*y*) and *k*(*x*,*y*), respectively. $\varphi \left(u,v\right)$ and $\varphi_{k}\left(u,v\right)$ are phase spectrums of *f*(*x*,*y*) and *k*(*x*,*y*), respectively.

For the amplitude spectrum analysis, we ignore the effect of the phase spectrum, so that the convolution kernels can be regarded as zero phase shift filtering and linear filtering can be performed on the SAR image. Typical zero-phase shift filters include low-pass filters, high-pass filters, band-stop filters, band-pass filters, and notch filters. The amplitude spectrum of each convolution kernel in the pre-trained CNN model can be compared with these typical filters, so as to analyze the frequency band where the features extracted are located by each convolution kernel. Specifically, ranges and directions of the pass frequency correspond to the physical significance of the extracted features. Thus, the role of the amplitude spectrum of the convolution kernel for feature extraction can be expressed more accurately.

In addition, according to the convolution theorem, the phase spectrum after convolution processing can be expressed as:

(12) $$\phi \left[F\left(u,v\right)\cdot K\left(u,v\right)\right]=\phi \left[\left|F\left(u,v\right)\right|e^{j\varphi \left(u,v\right)}\cdot \left|K\left(u,v\right)\right|e^{j\varphi_{k}\left(u,v\right)}\right]=\varphi \left(u,v\right)+\varphi_{k}\left(u,v\right),$$

where ϕ represents the processing of taking the phase spectrum. It is obvious that the phase spectrum of the convolution feature map is the sum of the phase spectrum of the feature map to be processed and the phase spectrum of the convolution kernel.

The phase spectrum mainly reflects the shape and position information of the target in the image. However, in general, it is difficult to obtain effective information by observing phase spectra directly. Therefore, the phase spectrum of images can be analyzed by using the reconstruction method based on the phase spectrum, so as to determine the image shape and position information carried by the phase spectrum [40]. Specifically, without changing the amplitude spectrum of the input image, the phase spectrums of both the input image and the convolution kernel are firstly summed point-to-point. Then, we can combine the phase spectrum after summation with the amplitude spectrum of the input image. Finally, the reconstructed image is obtained through 2D-IDF processing. It is clear that the reconstructed image is different from the input image only in the phase spectrum. This difference is caused by the phase spectrum of the convolution kernel. Therefore, the effect of the phase spectrum can be evaluated by obtaining the correlation between reconstructed images and input ones.

### 4.2. Actication Processing

In fact, the data may not be fitted by a linear function. Therefore, it is difficult to achieve accurate fitting results by using linear functions. In order to improve the mapping ability of the CNN, an activation function is introduced. The typical activation functions of the CNN include Sigmoid [41], Tanh [42], ReLU [43], etc.

(13) $$\mathrm{Sigmoid}\left(x\right)=\frac{1}{1+e^{-x}},$$

(14) $$Tanh\left(x\right)=\frac{1-e^{-2x}}{1+e^{-2x}},$$

(15) ReLUx=max0,x,

where *x* is the amplitude of each pixel of the feature map to be processed. From Equations (13) and (14), Sigmoid and Tanh are monotone-increasing functions. Namely, the relative size of the output is the same as that of the input. According to the universal approximation theorem in neural networks, the Sigmoid function is used to approximate the step function. That is, the step function can be decomposed into several linear combinations of simple S-type functions. Then, the given complicated function can be approximated with arbitrary precision. Here, the step function can be shown as:

(16) $$h_{a}\left(x\right)=\left\{\begin{array}{c} 1,x\geq a\\ 0,x<a \end{array}\right.,$$

where $h_{a}\left(x\right)$ is the step function and a∈ℝ represents the skip position. If $f\left(x\right)$ is a Sigmoid function and $h_{a}\left(x\right)$ is a step function that skips at a position, a sequence of S-type functions used to approximate $f\left(x\right)$ will be shown as:

(17) $$S_{n}\left(x;a\right)=f\left(n\left(x-a\right)\right)=\frac{1}{1+e^{-n\left(x-a\right)}},$$

If ∀ε>0, ∃N>0, and n>N, then:

(18) $$d\left(S_{n}\left(x;a\right),h_{a}\left(x\right)\right)=\int_{-\infty }^{+\infty }\left|f\left(x\right)-g\left(x\right)\right|dx<\varepsilon ,$$

**Figure 4:**
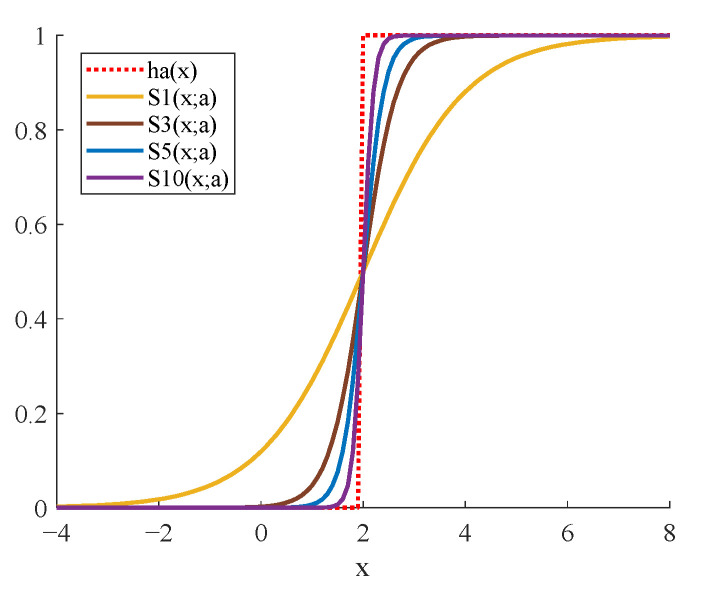
The fitting effect of Sigmoid to the step function.

Suppose a=2 in Equation (17), if n=1, n=3, n=5, and n=10, respectively, the differences between $S_{n}\left(x;a\right)$ and $h_{a}\left(x\right)$ are shown in Figure 4. It is obvious that with the gradual increase in *n*, the fitting effect becomes better. In the CNN, the Sigmoid processing of the feature maps can be understood as the approximate step processing and the normalization to the 0–1 interval. In addition, the Tanh function can be obtained by a linear transformation of the Sigmoid function. Therefore, the role of Tanh is similar to that of Sigmoid, both of which are an approximate processing of the step function.

To overcome the gradient disappearance problem of Sigmoid, ReLU is put forward. When the system meets certain assumptions, the output of the nonlinear link in the system under the action of the sinusoidal signal can be approximated by the first harmonic component. Therefore, the approximate equivalent frequency characteristic of the nonlinear processing is derived, namely, the description function. Then, the nonlinear system is approximately equivalent to a linear one. Furthermore, the frequency generation of the linear system theory can be used to analyze the nonlinear processing in the frequency domain. The ratio of the fundamental wave in the input and output of the nonlinear processing *N* is called the description function, which is used to represent the characteristics of *N*. It can be found by deduction when a sinusoidal signal is fed to the ReLU activation function, and its output fundamental wave is also a sinusoidal quantity of the same frequency. Moreover, its amplitude and phase become half of the input signal.

### 4.3. Full Connection

In classical CNN models, there is usually one fully connected layer or multiple ones. Taking AlexNet and VGG16 as examples, there are three full connection layers. Without considering the nonlinear activation processing, the relationship between the input and output of the fully connected layers can be expressed as:

(19) $$\begin{array}{cc} S_{n}\left(x;a\right) & =f\left(n\left(x-a\right)\right)=\frac{1}{1+e^{-n\left(x-a\right)}}\left[\begin{array}{c} y_{1}\\ y_{2}\\ \cdots \\ y_{m_{3}} \end{array}\right]\\ & =\left[\begin{array}{ccccc} w_{1,1}^{3} & w_{1,2}^{3} & \cdots & w_{1,n_{3}}^{3} & b_{1}^{3}\\ w_{2,1}^{3} & w_{2,2}^{3} & \cdots & w_{2,n_{3}}^{3} & b_{2}^{3}\\ \cdots & \cdots & \cdots & \cdots & \cdots \\ w_{m_{3},1}^{3} & w_{m_{3},2}^{3} & \cdots & w_{m_{3},n_{3}}^{3} & b_{m_{3}}^{3} \end{array}\right]\left[\begin{array}{ccccc} w_{1,1}^{2} & w_{1,2}^{2} & \cdots & w_{1,n_{2}}^{2} & b_{1}^{2}\\ w_{2,1}^{2} & w_{2,2}^{2} & \cdots & w_{2,n_{2}}^{2} & b_{2}^{2}\\ \cdots & \cdots & \cdots & \cdots & \cdots \\ w_{m_{2},1}^{2} & w_{m_{2},2}^{2} & \cdots & w_{m_{2},n_{2}}^{2} & b_{m_{2}}^{2}\\ 0 & 0 & 0 & 0 & 1 \end{array}\right]\left[\begin{array}{ccccc} w_{1,1}^{1} & w_{1,2}^{1} & \cdots & w_{1,n_{1}}^{1} & b_{1}^{1}\\ w_{2,1}^{1} & w_{2,2}^{1} & \cdots & w_{2,n_{1}}^{1} & b_{2}^{1}\\ \cdots & \cdots & \cdots & \cdots & \cdots \\ w_{m_{1},1}^{1} & w_{m_{1},2}^{1} & \cdots & w_{m_{1},n_{1}}^{1} & b_{m_{1}}^{1}\\ 0 & 0 & 0 & 0 & 1 \end{array}\right]\left[\begin{array}{c} x_{1}^{1}\\ x_{2}^{1}\\ \cdots \\ x_{n_{1}}^{1}\\ 1 \end{array}\right],\\ & =\left[\begin{array}{llll} \sum_{j=1}^{n_{2}}\sum_{i=1}^{n_{3}}w_{1,i}^{3}w_{i,j}^{2}w_{j,1}^{1} & \cdots & \sum_{j=1}^{n_{2}}\sum_{i=1}^{n_{3}}w_{1,i}^{3}w_{i,j}^{2}w_{j,n_{1}}^{1} & \sum_{j=1}^{n_{2}}\sum_{i=1}^{n_{3}}w_{1,i}^{3}w_{i,j}^{2}b_{j}^{1}+\sum_{i=1}^{n_{3}}w_{1,i}^{3}b_{i}^{2}+b_{1}^{3}\\ \sum_{j=1}^{n_{2}}\sum_{i=1}^{n_{3}}w_{2,i}^{3}w_{i,j}^{2}w_{j,1}^{1} & \cdots & \sum_{j=1}^{n_{2}}\sum_{i=1}^{n_{3}}w_{2,i}^{3}w_{i,j}^{2}w_{j,n_{1}}^{1} & \sum_{j=1}^{n_{2}}\sum_{i=1}^{n_{3}}w_{2,i}^{3}w_{i,j}^{2}b_{j}^{1}+\sum_{i=1}^{n_{3}}w_{2,i}^{3}b_{i}^{2}+b_{2}^{3}\\ \cdots & \cdots & \cdots & \cdots \\ \sum_{j=1}^{n_{2}}\sum_{i=1}^{n_{3}}w_{m_{3},i}^{3}w_{i,j}^{2}w_{j,1}^{1} & \cdots & \sum_{j=1}^{n_{2}}\sum_{i=1}^{n_{3}}w_{m_{3},i}^{3}w_{i,j}^{2}w_{j,n_{1}}^{1} & \sum_{j=1}^{n_{2}}\sum_{i=1}^{n_{3}}w_{m_{3},i}^{3}w_{i,j}^{2}b_{j}^{1}+\sum_{i=1}^{n_{3}}w_{m_{3},i}^{3}b_{i}^{2}+b_{m_{3}}^{3} \end{array}\right]\left[\begin{array}{c} x_{1}^{1}\\ x_{2}^{1}\\ \cdots \\ x_{n_{1}}^{1}\\ 1 \end{array}\right] \end{array}$$

where $w_{i,j}^{k}$ is the weight in position (*i*, *j*) of the kth layer; $b_{i}^{k}$ is the bias in position *i* of the *k*th layer; ${\left[x_{1}^{1},x_{2}^{1},\cdots ,x_{n_{1}}^{1},1\right]}^{T}$ is the input feature vector; ${\left[y_{1},y_{2},\cdots ,y_{m_{3}}\right]}^{T}$ is the output of the last fully connected layer. According to Equation (19), the output, ${\left[y_{1}^{3},y_{2}^{3},\cdots ,y_{m_{3}}^{3}\right]}^{T}$, can be obtained by multiplying the coefficient matrix by the input, ${\left[x_{1}^{1},x_{2}^{1},\cdots ,x_{n_{1}}^{1}\right]}^{T}$. Moreover, the coefficient matrix is the linear combination of weights and biases. Simplifying Equation (19), the expression for matrix multiplication is shown as:

(20) $$y_{k}={\left[\begin{array}{c} \sum_{j=1}^{n_{2}}\sum_{i=1}^{n_{3}}w_{k,i}^{3}w_{i,j}^{2}w_{j,1}^{1}\\ \cdots \\ \sum_{j=1}^{n_{2}}\sum_{i=1}^{n_{3}}w_{k,i}^{3}w_{i,j}^{2}w_{j,n_{1}}^{1}\\ \sum_{j=1}^{n_{2}}\sum_{i=1}^{n_{3}}w_{k,i}^{3}w_{i,j}^{2}b_{j}^{1}+\sum_{i=1}^{n_{3}}w_{k,i}^{3}b_{i}^{2}+b_{1}^{3} \end{array}\right]}^{T}\left[\begin{array}{c} x_{1}^{1}\\ x_{2}^{1}\\ \cdots \\ x_{n_{1}}^{1}\\ 1 \end{array}\right]={\vec{A}}^{T}\vec{X},$$

where $y_{k}$ is the *k*th node of the output, which can be expressed by the product of the coefficient vector ${\vec{A}}^{T}$ and the input vector $\vec{X}$. The multiplication of two vectors can be expressed as:

(21) $${\vec{A}}^{T}\vec{X}=\left|{\vec{A}}^{T}\right|\cdot \left|\vec{X}\right|\cdot \cos\theta ,$$

where θ is the angle between ${\vec{A}}^{T}$ and $\vec{X}$. For the pre-trained CNN model, the weights and biases of the full connection have been fixed. Hence, in the case of linear processing, the factors affecting the output of the full connection are the modulus value of the input and the angle between the input and the weight vector. When a certain feature vector is input in fully connected layers of the pre-trained CNN model, the category is confirmed by the angle between it and the different weight vectors.

## 5. Experiments and Discussion

**Figure 5:**
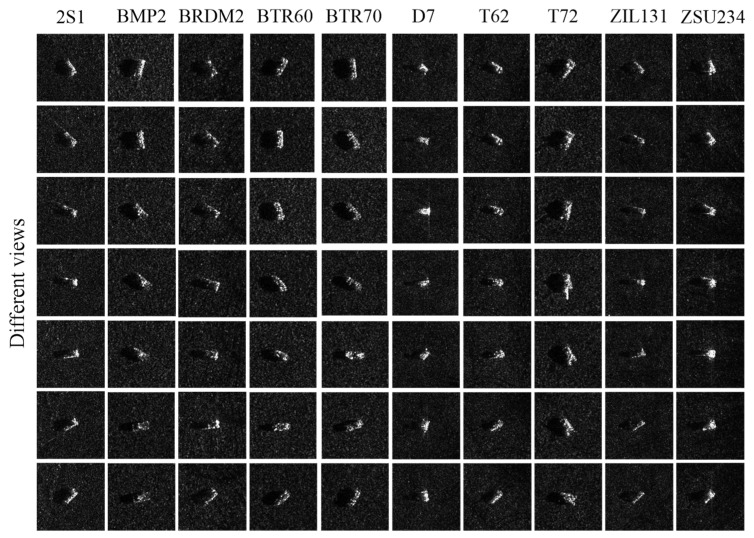
Different categories of targets in different views.

In this part, we use SAR images in the open MSTAR dataset to train the four classical models mentioned above. In the MSTAR dataset, according to different pitch angles, data quantities corresponding to 10 categories of targets are sorted out, respectively, as shown in Table 2. Here, the observation angle of each category of target varies from 0° to 180°. For the 10 categories of targets, imaging results under different view angles are presented, respectively, as shown in Figure 5. Consistent with the existing outcomes [44,45], SAR images with a pitch angle of 17° are taken as the training data. In the meantime, the four classical CNN models are trained by these images. Then, the pre-trained CNN models are taken as the experimental analysis object of this paper. Given this, it is analyzed whether the four classical pre-trained CNN models for SAR target recognition can effectively utilize these characteristics of the SAR image in this part, i.e., speckle noise and multi-view characteristics.

### 5.1. Convolution Processing

**Figure 6:**
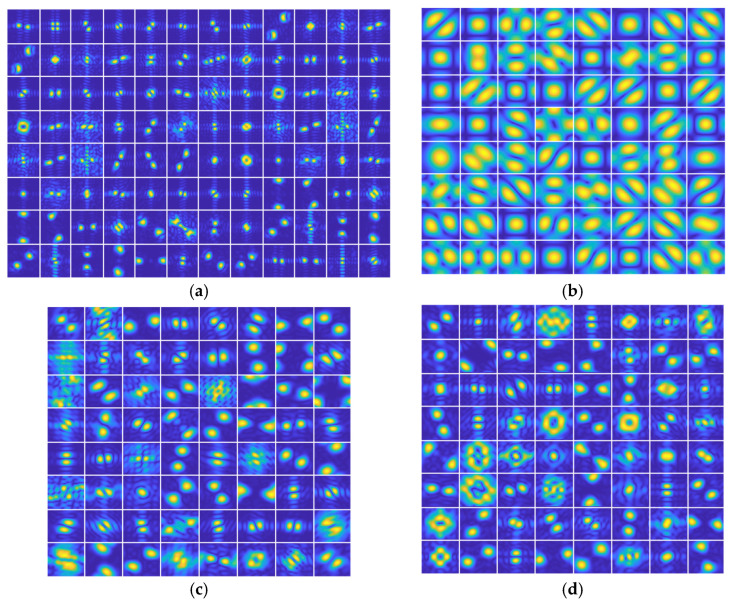
The amplitude spectrums of the convolution kernels in the first convolution layer of four classical pre-trained CNN models: (**a**) AlexNet; (**b**) VGG16; (**c**) GoogLeNet; (**d**) ResNet-50.

Firstly, the amplitude spectrum analysis is carried out. The amplitude spectrums of the convolution kernels in the first convolution layer of the four classical pre-trained CNN models is shown in Figure 6. Because of the same input of three channels of SAR images, the kernels are similar. We just show the one channel result. According to Section 4.1, the frequency range of extracted features can be displayed by the amplitude spectrum of the kernels. Hence, we can find that in the first convolution layer, the four classical pre-trained CNN models mainly extract the middle and high-frequency band information of the SAR image with direction, based on Figure 6. Here, many convolution kernels in VGG16 approximate the low pass filters. Namely, this model focuses on the general information extraction of SAR images in the first layer. In addition, most convolution kernels of the three pre-trained CNN models belong to the notch filter form. It can be found that the directions of the extracted features are not the same, which is consistent with the multi-view characteristic of the SAR images. Since the training samples we input are collected for different targets in different perspectives, more direction information needs to be extracted. Hence, the processing rationality of the first convolution layer is verified to a certain extent.

**Figure 7:**
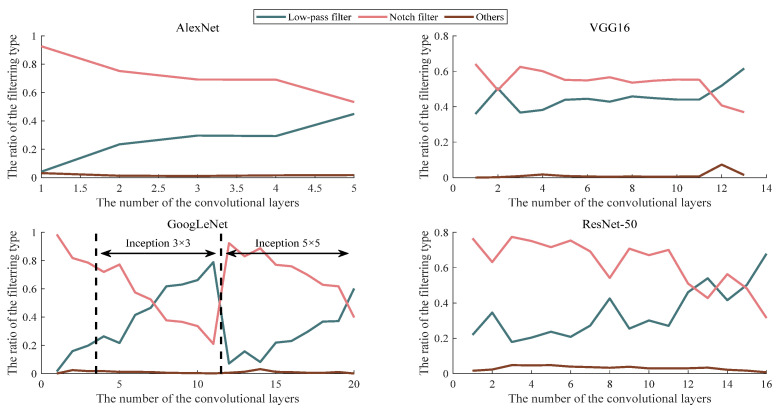
The proportional change of the filter types is presented by the amplitude spectrum of the convolution kernel with the deepening of the convolution layers.

Furthermore, the proportion of convolution kernel types in each convolution layer is statistically analyzed, except for 1 × 1 convolution kernels. The statistical result is shown in Figure 7. It is worth noting that there are some kernels with 3 × 3 and 5 × 5 parallel connections in the “Inception” module of GoogLeNet. To clarify the case of proportion variations with the number of layers, the proportions of 3 × 3 kernels are shown in the middle region and 5 × 5 ones are on the right side of the dotted line in Figure 7.

According to Figure 7, no matter how deep the number of convolution layers is, the convolution kernels in the convolution layer of the four classical CNN models belong to the low-pass filter or notch filter type in most cases. Moreover, the overall tendency is that with the deepening of the convolution layers, the proportion of the convolution kernels belonging to the notch-filter type gradually decreases, while the proportion of the low-pass filter increases. Especially in VGG16, GoogLeNet, and ResNet-50, the proportion of the low-pass filters in the deep convolution layers are higher than that of the notch filters. This phenomenon indicates that these pre-trained CNN models mainly extract the middle and high-frequency information in the low-level convolutional layer, which can be understood as extracting the contour information of a certain direction in the feature maps, respectively. In the deep convolution layers, the general information is extracted in the feature maps. This phenomenon corresponds to the size decrease of the SAR images and the reduction in target details in the feature maps after multi-layer processing. For the feature maps with less detailed information, the general features can be obtained by low-frequency filtering. It is more reasonable to take this as the key to the decision of some specific direction of the detailed information extraction again.

**Figure 8:**
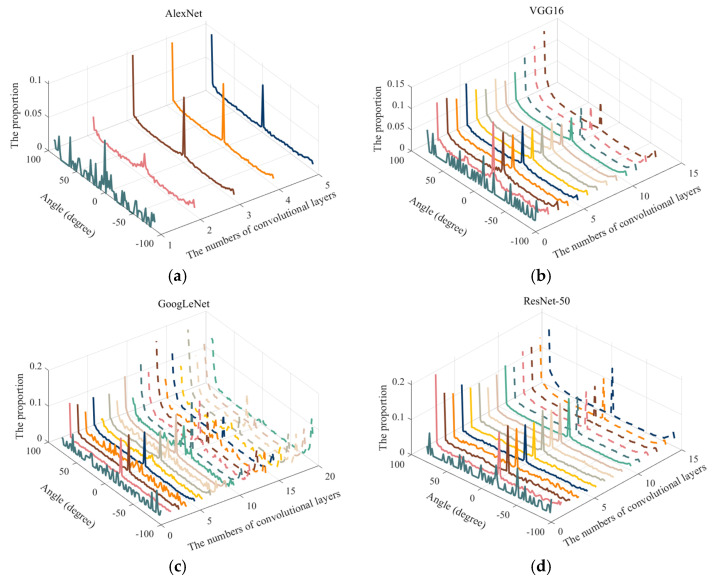
The angle distribution of features extracted from convolution kernels of notch filter type: (**a**) AlexNet; (**b**) VGG16; (**c**) GoogLeNet; (**d**) ResNet-50.

**Figure 9:**
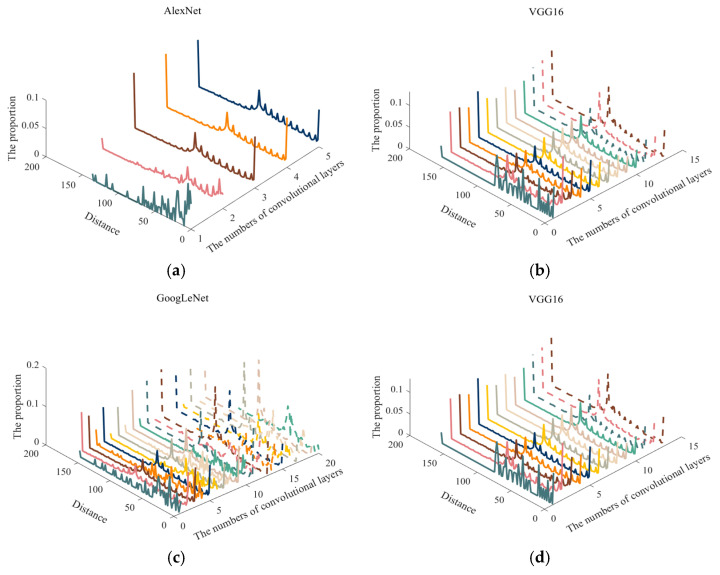
The distance distribution of features extracted from convolution kernels of notch filter type: (**a**) AlexNet; (**b**) VGG16; (**c**) GoogLeNet; (**d**) ResNet-50.

According to Figure 7, in most convolution layers of the four classical CNN models, the proportion of convolution kernels belonging to the notch filter is more than 0.5. In addition, since the notch filter can extract the features in specific directions and frequency bands, the distribution statistic of the filtering direction and the passing frequency range of the convolution kernel in each layer of the four classical CNN models is assessed. The results are shown in Figure 8 and Figure 9, respectively. Here, the “Angle” and “Distance” present direction and range, respectively.

According to Figure 9, the pass frequency ranges of the first convolution layers are mainly low and medium frequency bands in four classical pre-trained CNN models. From the second convolution layer to the last one, the frequency bands selected by the notch filters are mainly concentrated near the fundamental frequency, near the highest frequency and near the middle frequency. The main reason for this phenomenon is that the processing objects of the first convolution layer are the raw SAR images, which itself present granulate by speckle noises. Therefore, in the first convolution layer, the pre-trained CNN models are more inclined to smooth SAR image processing. In the meantime, the passing frequency bands are low and medium, which can exactly meet the requirement. From the second layer to the last one, general and detailed information needs to be extracted, so it is reasonable to concentrate near the fundamental and highest frequencies. In addition, the proportion near the middle band is relatively high, which proves that the four models also pay attention to the acquisition of some intermediate layer information of the feature maps.

From the above analysis, the role of the amplitude spectrum of the convolution kernels is verified. The amplitude spectrum influences the direction and frequency range of the extracted features. In other words, the analysis of the convolution kernels amplitude spectrum can qualitatively interpret the rationality of the convolution processing of the pre-trained CNN.

**Figure 10:**
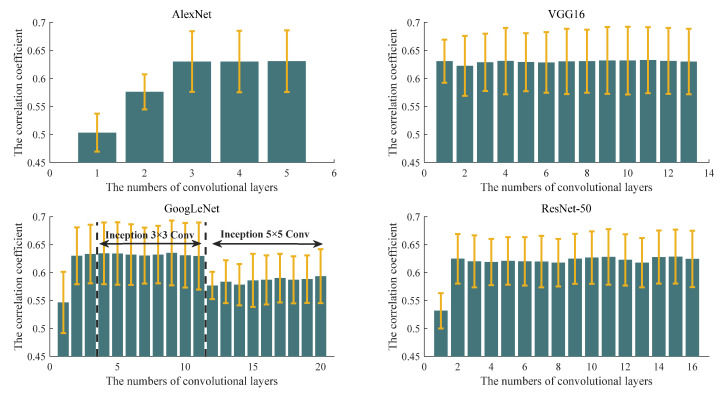
The mean and variance of the correlation coefficient in different layers.

To evaluate the effect of the phase spectrums of convolution kernels, the correlation coefficients between input images and reconstruction results are shown in Figure 10. According to Equation (7), we can find that the frequency spectrum of image *f*(*x*, *y*) is affected by the amplitude and phase spectrums. Here, the phase spectrum of the processing result is the sum of the phase spectrum of the input image and the phase spectrum of the convolution kernel. In this part, to analyze the function of the phase spectrum of the convolution kernels, we first reconstruct images without an amplitude spectrum effect as:

(22) $$f_{re}\left(x,y\right)=F^{-1}\left\{\left|F\left(u,v\right)\right|\cdot e^{j\left[\varphi \left(u,v\right)+\varphi_{k}\left(u,v\right)\right]}\right\},$$

It is obvious that, compared with input image *f*(*x*, *y*), the reconstruction result has a different phase spectrum, but the amplitude spectrums are same. Hence, we can evaluate the phase spectrum role of convolution kernels by correlation coefficients between *f*(*x*, *y*) and *f_re_*(*x*, *y*). The bigger the correlation coefficients, the lighter the role of the phase spectrum. In Figure 10, we show the mean and variance of the correlation coefficient in different layers. The variation trend of the phase spectrum of the convolution kernel with the deepening of the convolution layer can be analyzed. It is obvious that in the first convolution layer of AlexNet, GoogLeNet, and ResNet-50, the phase spectrums of the convolution kernels play an important role. As described in Section 4.1, the phase spectrums of the convolution kernels have an obvious influence on the shape of the target in the raw input SAR image, which is also a manifestation of shape information extraction. However, VGG16 is the different one, whose correlation coefficient mean values are basically maintained around 0.63, indicating that the phase spectrum is not the main effect. In addition, the larger the variance, the wider the distribution range of the correlation coefficients. It can be seen that the distributions in the four pre-trained CNN models are relatively concentrated in the low convolutional layers, while there are obvious deviations in the high layers. This indicates that the phase spectrums of the convolution kernels have a similar effect in the low convolution layers, but the effect difference is obvious in the high convolution layers. This is because the processing object is very different from the raw SAR images in the high layers. For the feature maps with shape information, the feature extraction should be enhanced, while for the ones without shape information, the feature extraction can be ignored. The extraction of the shape information represents the role of the phase spectrums of the convolution kernels, which can qualitatively prove the rationality of the pre-trained CNN model.

### 5.2. Full Connection Processing

**Figure 11:**
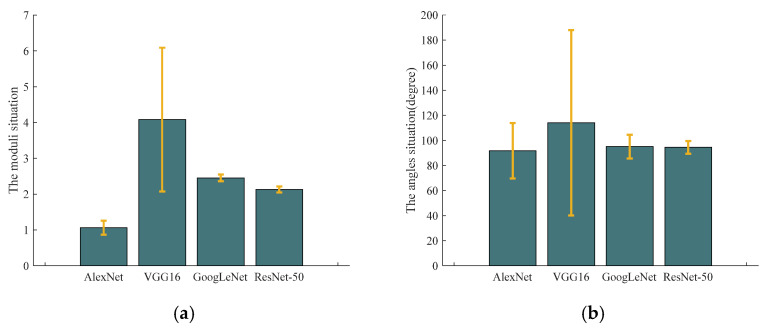
The means and variances of weight vector moduli and angles between the feature vectors and each of the column vectors for the four pre-trained CNN models: (**a**) the moduli; (**b**) the angles.

The same SAR image is input into four pre-trained CNN models to obtain feature vectors for input in the fully connected layer, respectively. Based on ignoring the nonlinear operation in the fully connected layer, the angles between the feature vectors and each column vector in the weight matrix of the fully connected layer are obtained, as well as the moduli of every column vector. The mean and variance are calculated, as shown in Figure 11. Although the mean of the modular value of the weight vector will directly affect the output value of the fully connected layer, the output of different target types is significantly different only when the variance is large. In the angle part, the variance value also affects the difference in output results for the different target types. Therefore, according to Figure 11, the pre-trained VGG16 model has the most obvious difference in the output of labels for different target types.

## 6. Conclusions

In this paper, we provide a process analysis method of the pre-trained CNN model used for SAR image target recognition. Firstly, given the inherent speckle noise and multi-view characteristics of SAR images, the features extracted by convolution processing are analyzed from the perspective of the frequency domain. Here, the influence of the amplitude spectrum on the direction and frequency range of extracted features is analyzed, as well as the shape information extracted from the phase spectrum. The rationality of the CNN feature extraction is qualitatively improved. Secondly, the activation functions are discussed. Among them, Sigmoid and Tanh functions can be transformed into each other through mathematical derivation, so the roles are similar. For the fully connected layer, in the case of ignoring the nonlinear processing, this part can be equivalent to the dot product of the input feature vector and different column vectors in the weight matrix, that is, the product of the moduli of the two vectors and the cosine of the angle between the two vectors. Hence, for the same input feature vector, the only factors affecting the recognition results are the moduli of the weight vectors and the angles between the two vectors. In the experiment, the analysis objects are pre-trained CNN models, i.e., AlexNet, VGG16, GoogLeNet, and ResNet50. The parameters are trained by ten categories of SAR target images in the public MSTAR dataset. Then, the processing of these pre-trained CNN models is analyzed by the proposed method. The conclusion is captured. Namely, aiming at the speckle noise characteristics of SAR images, the four models mainly extract the low and medium frequency features in the first layer of convolution processing, that is, to achieve the SAR images smoothing and effectively reduce the effect of granular noise. In addition, given the multi-view characteristics of the SAR images, the angle information extracted by the convolutional layers during feature extraction is scattered. In other words, features of different perspectives can be extracted. It is qualitatively proven that the four pre-trained CNN models have adaptability to SAR images. Moreover, through the analysis of the full connection, it can be found that the weight parameters in the fully connected layers of the VGG-16 are more conducive to distinguishing different categories of targets in the SAR images. In summary, this study provides a demonstration role for the process analysis of the pre-trained CNN model used for SAR target recognition.

## Figures and Tables

**Table 1:** Comparison of the recognition accuracy of four classical CNN models for SAR images with different qualities.

	AlexNet	VGG16	GoogLeNet	ResNet-50
*L* = 0.2	0.1129	0.1142	0.1129	0.1125
*L* = 1	0.1146	0.1237	0.1303	0.1401
*L* = 2	0.2187	0.4035	0.2523	0.2593
*L* = 3	0.5941	0.6002	0.3636	0.3603
*L* = 5	0.6950	0.7152	0.6026	0.7028
*L* = 8	0.8990	0.9221	0.8458	0.8545
*L* = 10	0.9426	0.9641	0.9413	0.9496
*L* -> +∞	0.9707	0.9703	0.9744	0.9726

**Table 2:** MSTAR dataset situations.

	Category	2S1	BMP2	BRDM2	BTR60	BTR70	D7	T62	T72	ZIL131	ZSU234
Pitch Angle											
17°		299	233	298	255	233	299	299	232	299	299
15°		274	195	274	195	196	274	273	196	274	274

## Data Availability

No new data were created or analyzed in this study. Data sharing is not applicable to this article.
